# *TaAPO-A1*, an ortholog of rice *ABERRANT PANICLE ORGANIZATION 1*, is associated with total spikelet number per spike in elite European hexaploid winter wheat (*Triticum aestivum* L.) varieties

**DOI:** 10.1038/s41598-019-50331-9

**Published:** 2019-09-25

**Authors:** Quddoos H. Muqaddasi, Jonathan Brassac, Ravi Koppolu, Jörg Plieske, Martin W. Ganal, Marion S. Röder

**Affiliations:** 1 Leibniz Institute of Plant Genetics and Crop Plant Research (IPK), Corrensstraße 3, D-06466 Stadt Seeland, OT Gatersleben, Germany; 2 TraitGenetics GmbH, Am Schwabeplan 1b, D-06466 Stadt Seeland, OT Gatersleben, Germany

**Keywords:** Agricultural genetics, Plant genetics

## Abstract

We dissected the genetic basis of total spikelet number (TSN) along with other traits, viz. spike length (SL) and flowering time (FT) in a panel of 518 elite European winter wheat varieties. Genome-wide association studies (GWAS) based on 39,908 SNP markers revealed highly significant quantitative trait loci (QTL) for TSN on chromosomes 2D, 7A, and 7B, for SL on 5A, and FT on 2D, with 2D-QTL being the functional marker for the gene *Ppd-D1*. The physical region of the 7A-QTL for TSN revealed the presence of a wheat ortholog (*TaAPO-A1*) to *APO1*–a rice gene that positively controls the spikelet number on the panicles. Interspecific analyses of the *TaAPO-A1* orthologs showed that it is a highly conserved gene important for floral development and present in a wide range of terrestrial plants. Intraspecific studies of the *TaAPO-A1* across wheat genotypes revealed a polymorphism in the conserved F-box domain, defining two haplotypes. A KASP marker developed on the polymorphic site showed a highly significant association of *TaAPO-A1* with TSN, explaining 23.2% of the total genotypic variance. Also, the *TaAPO-A1* alleles showed weak but significant differences for SL and grain yield. Our results demonstrate the importance of wheat sequence resources to identify candidate genes for important traits based on genetic analyses.

## Introduction

The wheat spike and its architecture are key components for improving grain yield. In the recent past, several genes controlling spike morphology have been investigated and described in temperate cereals^1,2^. Most spike morphological traits in wheat such as spike length and spikelet number behave as quantitative traits, and various QTL and association studies have recently been published^3–8^. High associations and prediction abilities for total and fertile spikelet number as well as spike length and grain yield were also reported^9^.

Only a few cloned genes for the trait number of spikelet pairs in wheat are available; among them is the *Q*-gene which played a major role in wheat domestication and encodes an *AP2* transcription factor^10^. The domesticated allele *Q* confers a free-threshing character, a sub-compact spike^11^, and is regulated by microRNA172^12^. Also, genes related to heading date are involved in spikelet meristem identity determination. For example, the photoperiodism gene *Ppd* was reported to influence spikelet primordia initiation^13^. Mutants of the *FLOWERING LOCUS T2* (*FT2*) in wheat showed a significant increase in the number of spikelets per spike with an extended spike development period accompanied by delayed heading time^14^. Moreover, *Ppd-1* and *FT* were reported as regulators of paired spikelet formation resulting in an increased number of grain-producing spikelets^15^. Mutants of the MADS-box genes, e.g., *VRN1* or *FUL2* showed an increased number of spikelets per spike, likely due to a delayed formation of the terminal spikelet^16^ and a putative ortholog to rice *MOC1* regulating axillary meristem initiation and outgrowth was associated with spikelet number per spike in wheat^17^.

The *ABERRANT PANICLE ORGANIZATION 1* (*APO1*) gene in rice was reported essential for regulating the inflorescence structure by controlling floral organ identity and floral determinacy^18,19^. *APO1* was shown important for maintaining proper inflorescence architecture and spikelet number by preventing precocious conversion of inflorescence meristem to spikelet meristems. On the molecular level, *APO1* encodes an F-box protein, an ortholog of *UNUSUAL FLORAL ORGAN* (*UFO*) in *Arabidopsis*, which regulates floral organ identity^19–22^. Four dominant mutants with elevated expression levels of *APO1* produced an increased number of spikelets by a delay in the programmed shift to spikelet formation. Ectopic overexpression of *APO1* resulted in increased meristem size caused by different rates of cell proliferation. It was concluded that the level of *APO1* activity regulates the inflorescence form through the control of meristematic cell proliferation^20^.

In the present study, we investigated the inheritance and genetic basis of total spikelet number (TSN) per spike, spike length and flowering time as component traits of grain yield in an elite European winter wheat panel. Our findings show the complex genetic architecture of the investigated traits, and that *TaAPO-A1*–an ortholog of rice *APO1*, which is vital for inflorescence development–is associated with the TSN determination in wheat. Intraspecific sequence analyses of *TaAPO-A1* revealed that polymorphisms were forming distinct haplotypes while interspecific studies showed the conserved nature of this gene across terrestrial plant species.

## Results

### Total spikelet number per spike is significantly correlated with spike length, flowering time, and grain yield

**Figure 1:**
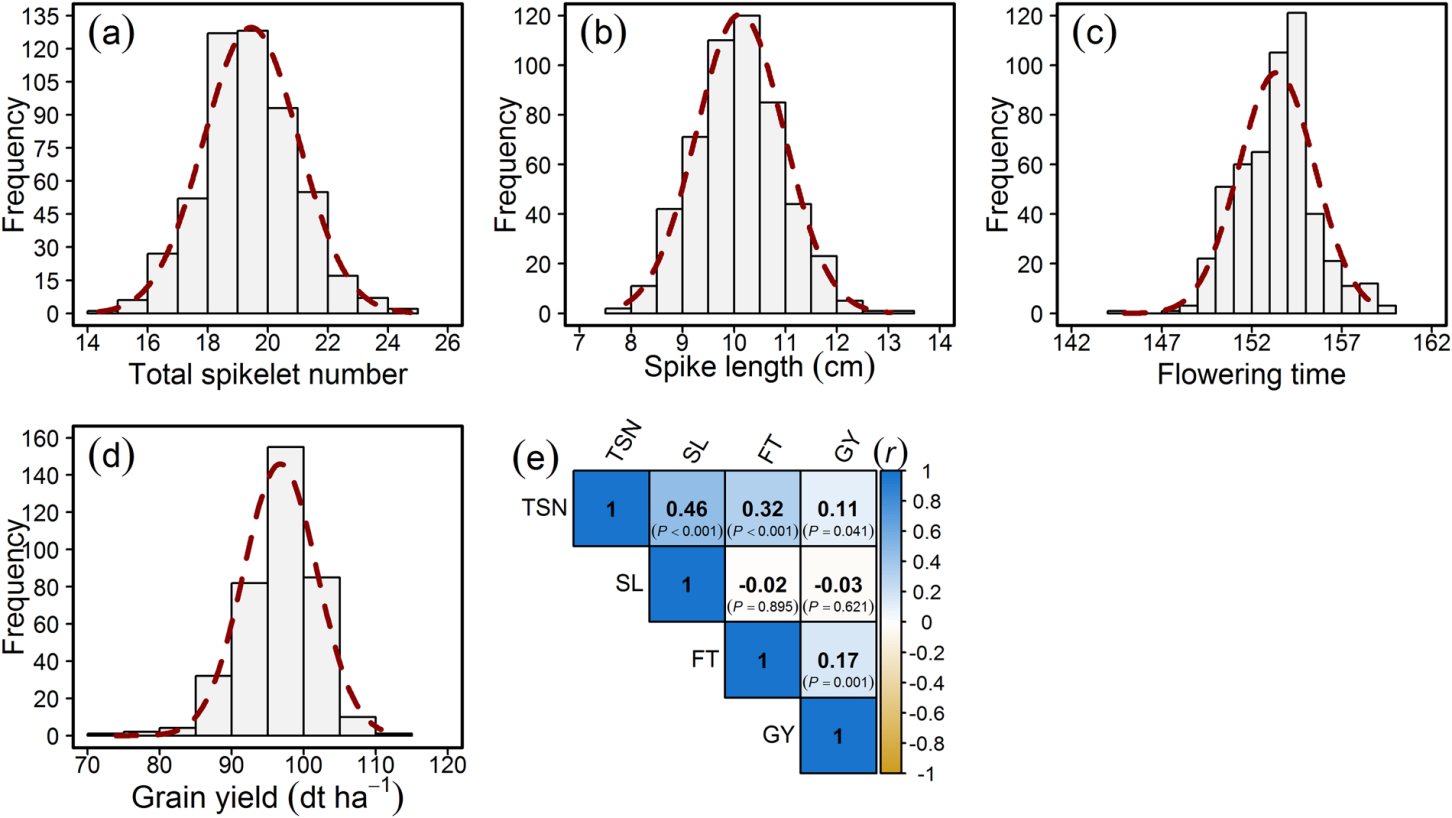
Distribution and correlation of the investigated traits in a panel of 518 elite European winter wheat varieties. Distribution of (**a**) Total spikelet number (TSN) per spike, (**b**) Spike length (SL), (**c**) Flowering time (FT), and (**d**) Grain yield (GY); (**e**) Pearson’s product moment correlation (*r*) among the investigated traits. *P*-value denotes the significance of the respective correlation.

The assessment of total spikelet number (TSN) per spike, spike length (SL), and flowering time (FT) were performed in the field trials on 518 elite European winter wheat varieties (including 15 spring type wheat varieties as an outgroup). The trait grain yield (GY) was assessed in multiple environment field trials on a subset (in total 372) of varieties in a previous study^23^. The best linear unbiased estimations (BLUEs) of all traits approximated normal distribution and showed wide variation (Fig. 1a–d; Table S1a). The ANOVA showed that genotypic (*σ*_*G*_^2^) and environmental (*σ*_*E*_^2^) variation was significantly (*P* < 0.001) larger than zero (Table 1). The broad-sense heritability estimates ranging from 0.68 to 0.89 indicated the good quality of the phenotypic data and its potential for use in genome-wide association studies (GWAS) to map the quantitative trait loci (QTL) underlying the traits (Table 1). We analyzed the Pearson’s product-moment correlation (*r*) among the best linear unbiased estimations (BLUEs) of the investigated traits, which revealed that TSN was positively and significantly correlated with SL, FT, and GY (Fig. 1e). The TSN and SL showed the highest correlation among the investigated traits (r = 0.46; P < 0.001) whereas SL showed almost a null correlation with FT and GY suggesting that FT augments GY mainly by influencing the TSN in wheat.

**Table 1:** Summary statistics of the investigated traits, namely total spikelet number (TSN) per spike, spike length (SL; cm), flowering time (FT), and grain yield (GY; dt ha^−1^).

Parameter	TSN	SL	FT	GY
Minimum	14.38	7.90	144.96	73.94
Mean	19.45	10.12	153.38	96.74
Maximum	24.75	13.05	159.61	110.71
*σ*_*G*_^2^	1.71^a^	0.50^a^	3.42^a^	22.89^a^
*σ*_*E*_^2^	1.75^a^	1.63^a^	6.30^a^	94.51^a^
*σ*_*e*_^2^	1.60	0.44	1.90	23.74
*H* ^2^	0.68	0.70	0.84	0.89
*nE*	2	2	3	8

*σ*_*G*_^2^ = genotypic variance; *σ*_*E*_^2^ = environmental variance; *σ*_*e*_^2^ = residual variance; *H*^2^ = broad-sense heritability; *nE* = number of environments; a = significant at < 0.001 probability level.

### High-density marker arrays reveal the absence of distinct sub-populations and sharp LD decay in European elite winter wheat

**Figure 2:**
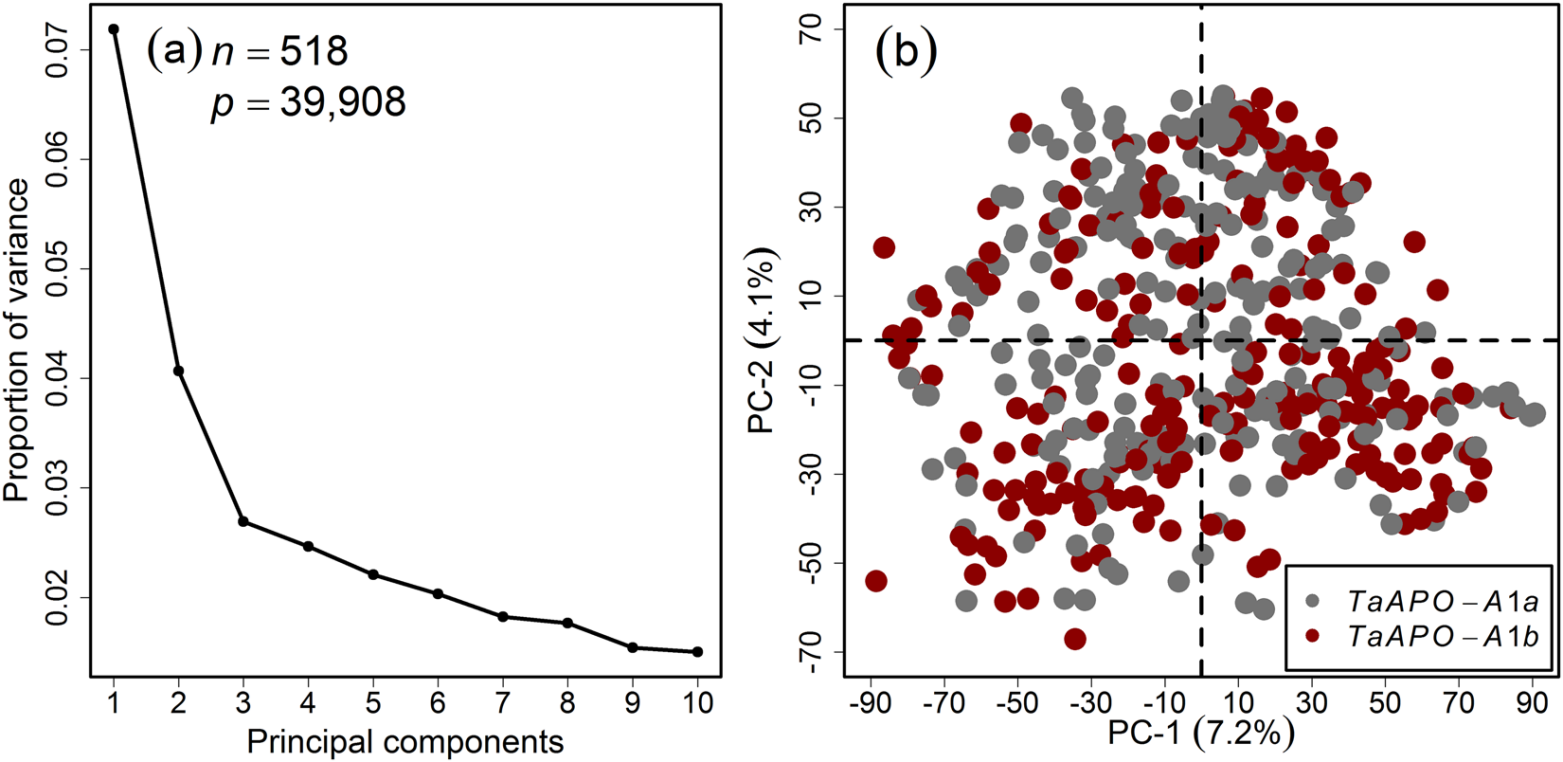
Principal component (PC) analysis on the wheat marker loci combined from the 35k and 90k single nucleotide polymorphism arrays. (**a**) Scree plot showing the first ten PCs and their corresponding proportion of variance, (**b**) Scatterplot showing the absence of pronounced sub-clustering among the varieties. Different colors represent the *TaAPO-A1* alleles. *n* and *p* denote the number of varieties and the marker genotypes used in the analysis, respectively.

The whole wheat panel was extensively genotyped with high-density SNP arrays and functional markers for the genes *Ppd-D1*, *Rht-B1*, *Rht-D1*, *Vrn-A1*, *Vrn-B1*, and *Vrn-D1*, which resulted in 39,908 high-quality markers. The population structure analyzed with marker genotypes by principal component (PC) analysis resulted in the absence of distinct sub-populations with the first two PCs representing only 11.3% of the variation (Fig. 2). The high familial relatedness and non-existence of distinct sub-populations were further supported by plotting a heat map of the genomic relationships among the wheat varieties (Fig. S1) and by the structure-like inference algorithm LEA, which resulted in the sub-populations being distinguished but with a slight entropy shift. The bar plots indicated admixed and weak sub-populations (Fig. S2).

**Figure 3:**
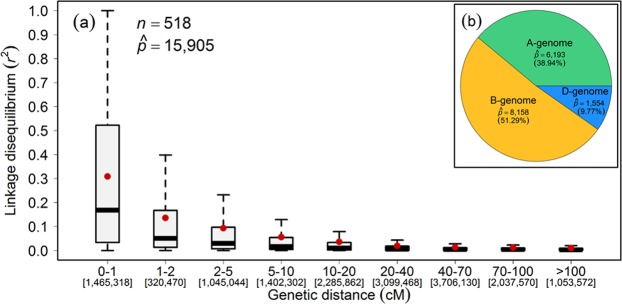
Genome-wide decay of linkage disequilibrium (LD; *r*^2^) as a function of genetic map distance (cM) between the marker loci in the population of European winter wheat varieties. (**a**) Boxplots represent the LD-decay, (**b**) Sub-genome-wise distribution of mapped marker loci. Red dots within the boxplots represent the mean. The numbers on second row of x-axis represent the number of marker-pairs present in the corresponding genetic distance. *n* and $\hat{p}$ denote the number of varieties and mapped marker loci, respectively.

Linkage disequilibrium (LD) between the marker genotypes determines the number of markers needed to perform GWAS. Genome-wide LD analysis was performed with the mapped marker genotypes which resulted in a rapid LD decay by increasing the genetic (cM) distances: first and third quantile dropped to 0.002 and 0.028, respectively, and the mean and median values equaled 0.051 and 0.008, respectively (Fig. 3a). The sub-genome-wise distribution of the markers varied: the highest number of markers mapped on B-genome, followed by A- and D-genomes (Fig. 3b). Although the whole panel was genotyped with state-of-the-art genotyping arrays, the sub-genome-wise distribution of marker genotypes suggests that the marker density could be improved especially for D-genome.

### GWAS identifies large-effect QTL for TSN on chromosome 7A in European winter wheat

**Figure 4:**
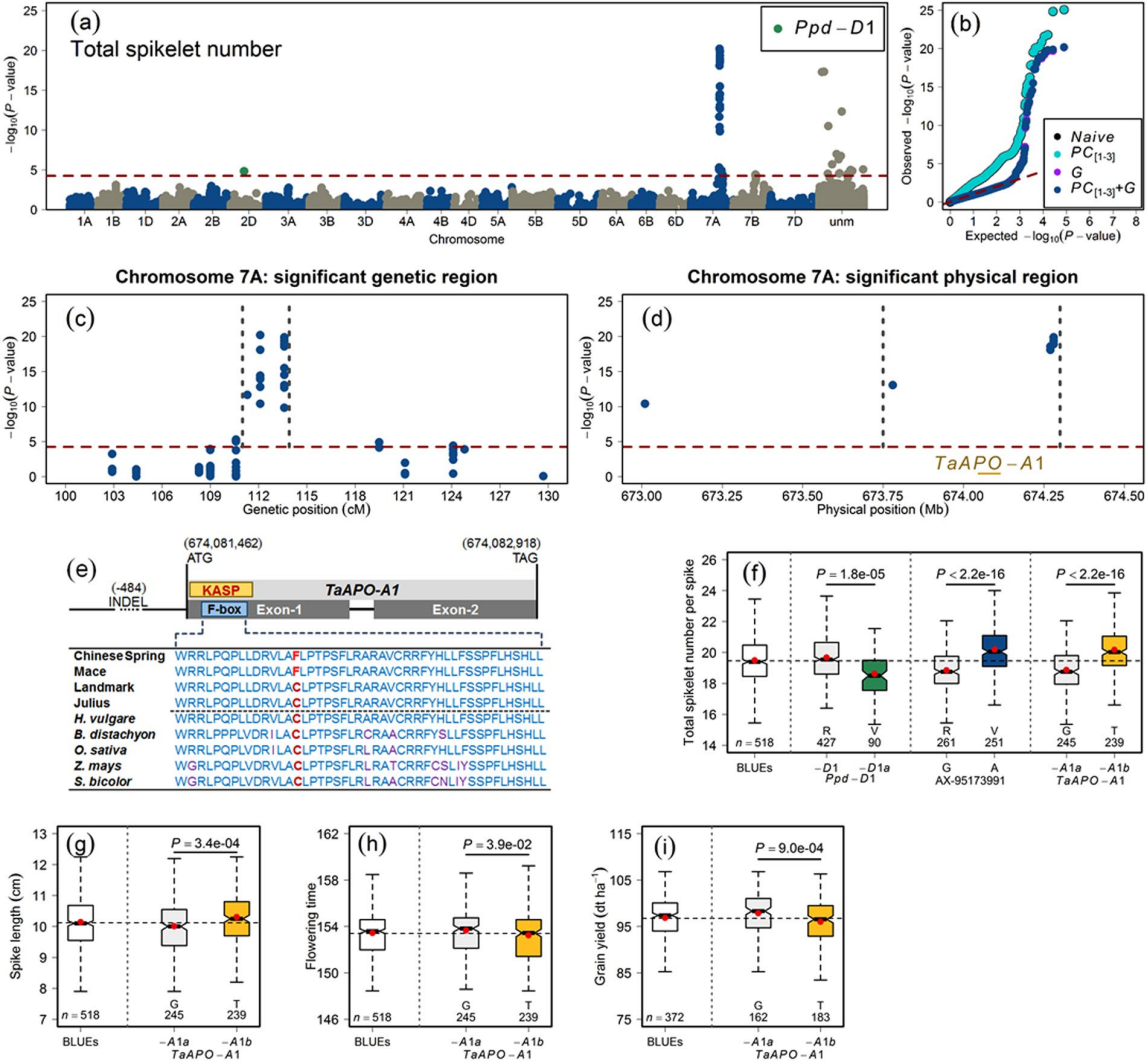
Summary of the genome-wide association studies (GWAS) of total spikelet number per spike in the population of 518 European winter wheat varieties. (**a**) Manhattan plot shows the distribution of marker significance −log_10_(*P* − value) along the chromosomes. The correction for population stratification and familial relatedness was performed by using the first three principal components (*PC*_[1–3]_) and an additive genomic relationship matrix (*G*) in a linear mixed-effect model. The red dashed line marks the multiple testing criteria of false discovery rate (FDR) <0.05, (**b**) Quantile-quantile plot showing the distribution of observed *versus* expected (red dashed line) −log_10_(*P* − value). The naïve model represents the GWAS without the correction of population structure, the *PC*_[1–3]_ model represents the population structure corrected with the first three *PCs*, the *G* model represents the familial relatedness corrected with a genomic relationship matrix, and the *PC*_[1–3]_ + *G* model represents the population structure and familial relatedness corrected with the first three *PCs* and the *G* matrix. The color code for different models is given in the figure legend, (**c**) Significant genetic region on chromosome 7A for TSN in wheat. The gray vertical dashed lines mark the highly significant genetic region, (**d**) Significant physical region on chromosome 7A for TSN in wheat. The gray vertical dashed lines mark the highly significant physical region, (**e**) Gene structure of the *TaAPO-A1*. The orange box represents the location of the KASP marker developed to exploit the variation in the F-box domain (highlighted in blue color). The horizontal line preceding the first exon depicts the promotor region harboring an INDEL and its corresponding position. The first four rows represent the F-box sequences of wheat varieties (courtesy: *The 10*+ *Wheat Genomes Project*) and the second five rows represent the F-box domain of closely related species viz. *Hordeum vulgare*, *Brachypodium distachyon*, *Oryza sativa*, *Zea mays*, and *Sorghum bicolor*. The non-synonymous mutation is highlighted in red color. The location of start and stop codons on chromosome 7A are given in the figure, (**f**) Allele-wise phenotypic distribution of the most significant markers and the KASP marker for *TaAPO-A1* associated with (**f**) TSN, (**g**) Spike length, (**h**) Flowering time, and (**i**) Grain yield. *P-value* denotes the significance value of the two-sided *t*-test used to compare the mean value of the marker alleles. In sub-figures (**f**) to (**i**), the first boxplots represent the distribution of the best linear unbiased estimations (BLUEs) for the respective trait.

Among the different GWAS models used in our study, we observed that the *PC*_[1–3]_ + *G* model could best control the spurious marker-trait associations (MTA). Our GWAS analyses identified QTL on chromosomes 2D, 7A, and 7B for TSN (Fig. 4a,b; Table S2a), for SL on chromosome 5A (Fig. S3, Table S2b), and for FT on chromosome 2D (Fig. S4; Table S2c). The QTL on chromosome 2D identified for TSN and FT was most likely the gene *Ppd-D1*. The photoperiod insensitive allele *Ppd-D1a* significantly reduced the TSN in wheat (Fig. 4a,f). The phenotypic data for GY were analyzed to investigate if there exists any significant correlation between the identified marker alleles and GY (Fig. 4i). The total proportion of genotypic variance (*p*_*G*_) imparted by the identified mapped QTL amounted to 65.44% for TSN, 15.15% for SL, and 31.58% for FT. A relatively low *p*_*G*_ explained for SL and FT is the result of the identification of only one mapped marker for each trait.

For TSN, we identified 43 MTA: one mapped to chromosome 2D (*Ppd-D1*), 24 on chromosome 7A, three on chromosome 7B, while 15 MTA were unmapped according to our mapping (ITMI) resources (Table S2a). We used additional consensus maps^24,25^ to assign the chromosomes to the 15 unmapped markers. By following this approach, we could assign 14 out of 15 unmapped markers (13 to chromosome 7A and one to 7B) whereas one marker was unmapped according to all mapping resources (Table S2a). Moreover, we analyzed the similarity between the chromosomal assignments of our mapped markers with other consensus maps. The chromosomal assignments of our mapped markers corresponded to other maps with a 100% congruence (Table S2a,b). However, although the chromosomes assigned to the SNPs are the same in all mapping resources, the genetic (cM) positions differ–a most likely reason is that the mapping populations used in different resources are dissimilar. Nevertheless, of interest is a large-effect QTL identified for TSN on chromosome 7A–for which the most significant marker *AX-95173991* is located at 112.10 cM and explained 25.70% of the total genotypic variance (Fig. 4a,b, Table S2a). This warrants, on the one hand, that the use of 7A-QTL would be beneficial for efficient marker-assisted selection. On the other hand, it made possible the further investigation of 7A-QTL at the physical sequence level to search for candidate genes.

### Significant physical region of chromosome 7A-QTL harbors *TaAPO-A1*–a putative candidate gene for TSN in wheat

The significant 7A-QTL genetic region for TSN spanned initially from 110.6 to 124.1 cM (Table S2a). We narrowed down the genetic region with the highly significant MTA with −log_10_(*P* − value) >10 within 2.3 cM starting from 111.3 to 113.6 cM (Fig. 4c). The alignment of marker sequences present within this most significant genetic region onto chromosome 7A revealed a physical region starting from 673.78 to 674.30-Mb (Fig. 4d) that harbored only ten genes (Table S3). The functional annotations of these ten genes revealed an interesting candidate gene *TraesCS7A01G481600*; (physical map position: 674,081,462–674,082,918-bp) with functional annotation as *Aberrant panicle organization 1* (*APO1*) *protein* (Table S3). The *APO1* in rice regulates inflorescence architecture and positively controls the total spikelet number by suppressing the precocious conversion of inflorescence meristems to spikelet meristems^18,19^.

### A KASP marker developed for the *TaAPO-A1* shows significant association with TSN in wheat varieties

**Figure 5:**
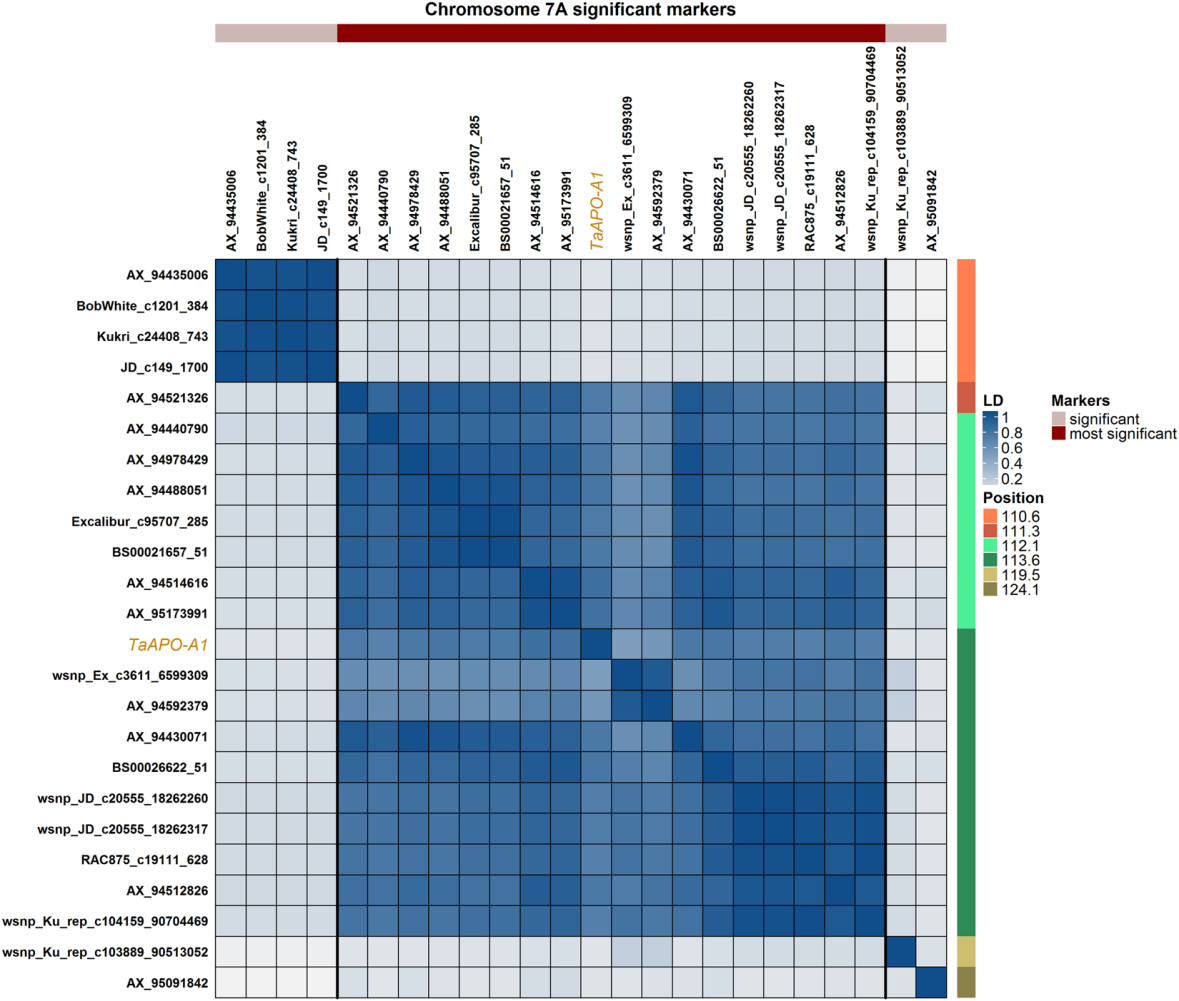
Pairwise linkage disequilibrium (LD; *r*^2^) among the marker loci (including the KASP marker for the gene *TaAPO-A1*) present in the significant genetic region of TSN on chromosome 7A in wheat. Based on the linkage blocks, markers are divided into two categories viz. significant, and most significant. The color key is given in the figure.

*TaAPO-A1* is a 1,457-bp long gene and, like *APO1* in rice, it has two exons separated by one intron (Fig. 4e). We investigated the variation of *TaAPO-A1* in ten wheat varieties which revealed two haplotypes–the sequences were taken from *The 10*+ *Wheat Genome Project* (Figs 4e and S6). The first exon harbors a highly conserved F-box domain of 46 amino acid residues across the wheat varieties and other species (Figs 4e, S6 and S7). Intraspecific sequence analysis of *TaAPO-A1* revealed a non-synonymous mutation in the F-box domain: out of ten wheat varieties, four (including Chinese Spring) harbored T while six had G allele. We developed a KASP marker for *TaAPO-A1* harboring this non-synonymous mutation in the F-box domain (Table S1b). The KASP marker for *TaAPO-A1* was highly significantly associated with TSN (Fig. 4f) and the marker alleles were evenly distributed in the variety panel (Fig. 2b; Table S1a). The second round of GWAS was performed by the *TaAPO-A1* KASP marker integrated into the original SNP matrix which further confirmed the significant association of *TaAPO-A1* with TSN, explaining 23.21% of the total genotypic variance (Fig. S5; Table S2a). The reference allele in the population (represented by *TaAPO-A1a*, with nucleotide G translating to cysteine) was present in 50.62% of the investigated varieties and resulted in an average TSN of 18.83, whereas the variant allele (represented by *TaAPO-A1b*, with nucleotide T translating to phenylalanine) was present in 49.38% of the varieties and revealed an average TSN of 20.13 (Fig. 4f, Table S1a). The analysis of local linkage disequilibrium performed with the markers present in the 7A-QTL genetic region and the KASP marker for *TaAPO-A1* showed that *TaAPO-A1* was in tight linkage with other markers (Fig. 5). Furthermore, we also observed a rather weak but significant association of the *TaAPO-A1* KASP marker alleles with SL, FT, and GY (Fig. 4g–i).

The single nucleotide substitution G (low TSN allele) to T (high TSN allele) in the conserved functional domain of TaAPO-A1 resulted in a non-synonymous amino acid substitution from cysteine (C) to phenylalanine (F). The amino acid cysteine appears to be well conserved across various grass species at this position potentially indicating the conservation of C residue across grasses. However, the SIFT (Sorting Intolerant from Tolerant) score^26^ analysis showed no potential deleterious effect from C to F substitution at this position (Table S4). We then looked at the promoter region of *TaAPO-A1* in ten genotypes from *The 10*+ *Wheat Genome Project* and identified a 115-bp INDEL (insertion-deletion) polymorphism at −484-bp upstream of the transcription start site of *TaAPO-A1*. Interestingly, the low TSN haplotype “G” (coding for cysteine) always had a deletion of 115-bp in the promoter, whereas the high TSN haplotype “T” (coding for phenylalanine) had 115-bp insertion. It, nevertheless, remains to be established via functional studies if this INDEL affects the transcription rate of *TaAPO-A1* contributing to the observed phenotypic differences for TSN in two haplogroups.

### Phylogenetic analyses show that TaAPO-A1, an ortholog of UFO in *Arabidopsis*, is conserved across terrestrial plant species

**Figure 6:**
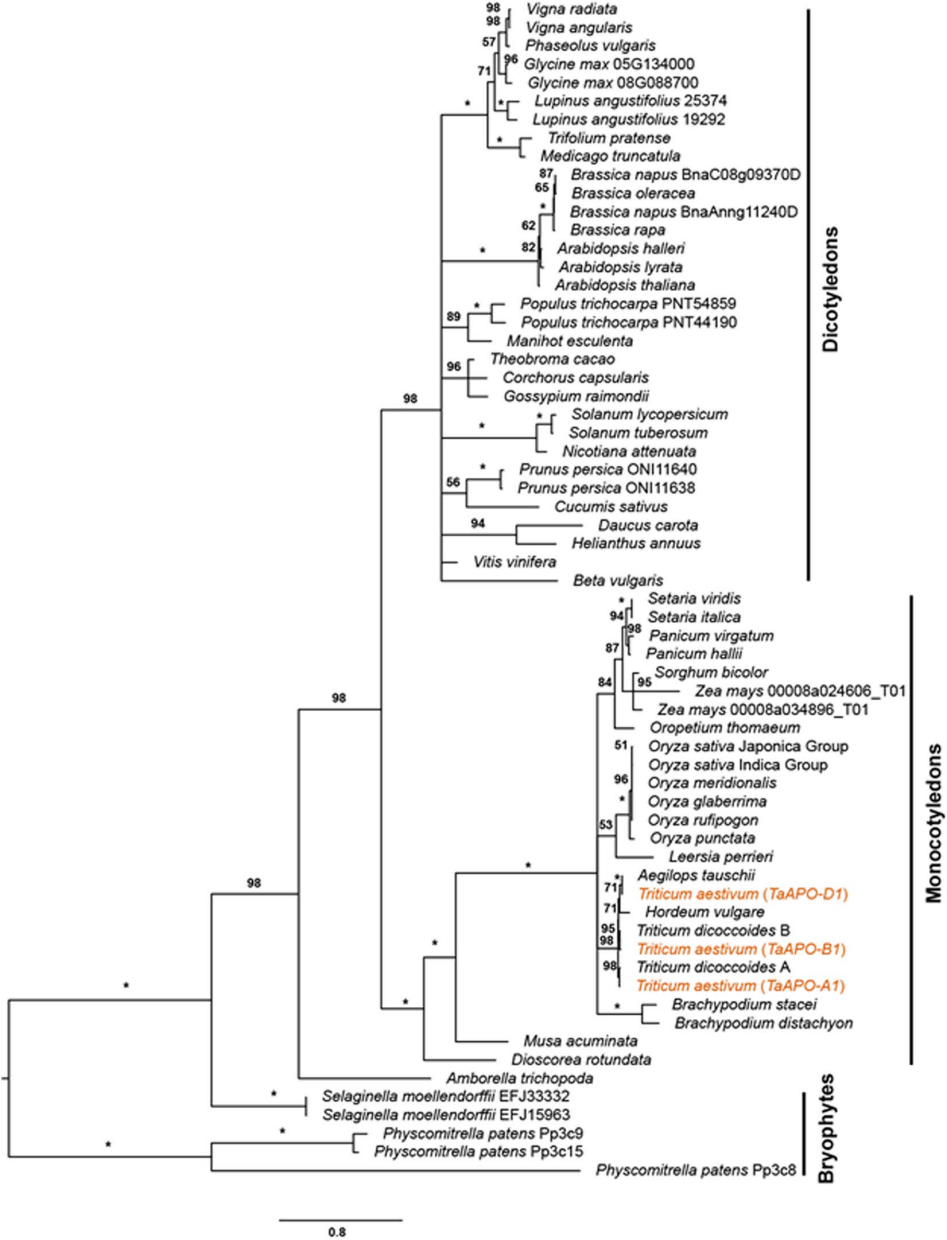
Maximum likelihood phylogenetic tree of TaAPO-A1 orthologous proteins across terrestrial plant species. Bootstrap values are indicated along the braches. Asterisks indicate >99% bootstrap values. The *TaAPO* homoeologs are highlighted in orange color. The bars on the right side indicate the major clades. The amino acid substitution scale is indicated at the bottom of the figure.

The BLAST search of TaAPO-A1 orthologs across diverse plant species from the EnsemblPlants and the protein databases Phytozome *v*12.1 retrieved 64 protein sequences from 37 genera (52 species, Table S5) including Bryophytes, monocotyledons, and eudicotyledons. The final alignment consisted of 670 positions. The obtained maximum likelihood (ML) topology reflects the evolution of terrestrial plants with *Amborella trichopoda* at the base of the two main clades, monocotyledons and eudicotyledons (Fig. 6). The protein is relatively well conserved as seen from the tiny branches especially within the grass tribe Triticeae, including *Triticum aestivum* and *Hordeum vulgare*, which diverged about ten million years ago (Ma)^27^ or even the Poaceae, whose most recent common ancestor probably occurred 50–75 Ma^28^.

## Discussion

### Exploiting significant, heritable genetic variation of TSN as well as a positive correlation with other traits can help to improve the grain yield in wheat

Grain yield (GY) improvement is considered as the top focus of virtually every wheat breeding program. However, an extremely complex genetic nature of GY often hampers its genetic improvement as it is the product of several yield components, e.g., the number of spikes per plant, grains per spike, thousand-grain weight. The number of grains per spike is the product of total spikelet number (TSN) per spike and spikelet fertility. Therefore, an essential consideration in wheat breeding has been to employ a reductionist approach, i.e., to exploit the information about the individual component traits–most of which are negatively associated with each other. In this study, we analyzed a winter wheat panel comprising 518 varieties for GY component traits such as TSN and spike length (SL) along with the flowering time (FT). The GY data based on previous studies were taken for comparison purposes^23^. In all observed traits, besides significant genetic variation, we observed a significant genotype-by-environment (year) interaction. Nevertheless, the broad-sense heritability estimates ranging from 0.68 to 0.89 suggested that genetic variation is heritable–an essential indicator of high selection response (Table 1). Similar heritability values for the investigated traits have been reported recently in other diverse mapping populations^4,6^.

In addition to significant genetic variation, TSN showed a positive and significant correlation with SL, FT, and GY (Fig. 1e). A relatively low correlation of TSN with GY in comparison to SL and FT may not be a true reflection of the relationship between TSN and GY since GY data was taken from a study that investigated different number of lines (372) in different sets of environments. In this set of varieties, the Chinese Spring allele coding for “T” was associated with an increase in TSN, but a decrease in GY. However, albeit being weak (which is by virtue of the extreme quantitative genetic nature of GY), TSN’s correlation with GY could help in selection and improve the genetic gains. Moreover, it should be noted that the genetic architecture of yield component traits *per se* is also important which means that if the component traits possess complex genetic architecture, the problem of grain yield improvement would be further compounded. Nevertheless, a reasonably high heritability value suggests that TSN is strongly genetically inherited and that the mapping of the underlying quantitative trait loci (QTL) would be efficient.

### High marker density governs the efficacy of genetic and physical mapping

The efficiency of genome-wide association studies (GWAS) depends on the size of the population and genetic diversity. Therefore, genome-wide marker density with many polymorphic sites is vital, and coupled with a sharp decline in linkage disequilibrium (LD) between the marker loci, it increases the GWAS resolution. In our study, the size of the population, high-density genotyping, and the use of stringent linear mixed-effect models warranted the genetic mapping of true marker-trait-associations (MTA). As noted in another study based on a subset of varieties, the absence of distinct sub-populations in this panel suggests that the European winter wheat varieties have been bred, by and large, from a narrow genetic base and with similar goals^29^ which is in line with other reports based on studies using similar genetic material but different marker platforms^30,31^.

To identify the candidate genes, high marker density in the genetic regions of the quantitative trait loci (QTL) is necessary since it helps to narrow down to the physical regions harboring the gene(s) underlying the trait. Moreover, since GWAS hinges on the principle that the markers work as proxies to the genes/QTL underlying the traits, a high density of markers in the QTL genetic region becomes vital for the success of fine mapping. In this study, we exploited this premise to identify a candidate gene physically.

### Physical mapping shows that *TaAPO-A1* is a likely candidate gene for TSN in wheat

Our GWAS analyses revealed a significant QTL for TSN on chromosome 7A, which explained ~25% of the total genotypic variance. Also, Würschum *et al*.^6^, recently reported a QTL for TSN on chromosome 7A in a similar type of elite winter wheat germplasm. Zhang *et al*.^17^, reported a putative *MOC1* ortholog to be associated with spikelet number, which is also located on chromosome 7A.

The strategy to investigate orthologous genes of rice with the known function was already successfully applied for various genes associated with grain size, grain weight, and yield in wheat^32–37^. The highly significant region of the detected TSN-QTL in our study corresponded to a physical interval of <1-Mb, containing a block of only ten genes, all in high LD (Fig. 5). Based on the functional annotations, the rice gene *ABERRANT PANICLE ORGANIZATION 1* (*APO1*), an ortholog of *Arabidopsis UFO*^18,19,21,22^, was considered as the most likely candidate gene and was named as *TaAPO-A1* in wheat. The functional analyses in both rice and *Arabidopsis* revealed that the F-box containing proteins are involved in the regulation and development of floral organs–more specifically, *APO1* in rice controls the number of spikelets per panicle by regulating the cell proliferation in meristems^20^. Recently, two independent studies based on GWAS and linkage mapping reported *APO1* as the best candidate gene affecting the TSN per spike in wheat^38,39^.

### Functional diversity among the orthologs of TaAPO-A1 reveals the conserved F-box domain

The availability of genomic data for several wheat varieties from *The 10*+ *Wheat Genome Project* allowed the investigation of the intraspecific diversity of *TaAPO-A1* gene. The *TaAPO-A1* contains two exons, each containing a SNP which causes an amino acid substitution. In the first exon, a T/G polymorphism at base 140 was related to the exchange of phenylalanine to cysteine, and in the second exon, at base 1,284, a G/A polymorphism mutated aspartic acid to asparagine (Fig. S6). It was possible to develop a functional KASP marker for the SNP in the first exon and screen the whole germplasm panel. Both alleles were present in almost identical frequencies with 49.38% of the varieties carrying the allele of Chinese Spring with nucleotide T (referred to as *TaAPO-A1b*) and 50.62% of the varieties carrying the G nucleotide (referred to as *TaAPO-A1a*). The Chinese Spring allele was strongly associated (*P* < 2.2e-16) with an increase in TSN and moderately associated with an increase in SL (*P* = 3.4e-04) and a decrease in GY (*P* = 9.0e-04) (Fig. 4f–i). For the B- and D-genomes, the orthologs of *TaAPO-A1* were related to the genes *TraesCS7B01G384000* and *TraesCS7D01G468700*. However, no MTA were discovered on these genomes. The identified *TaAPO-A1* variants reflect natural allelic diversity with mild phenotypic effects, which is beneficial for practical breeding.

The presence of *TaAPO-A1* orthologs in a wide range of plants including Bryophytes, monocotyledons and eudicotyledons suggests a central role of this gene class in the evolution and development of terrestrial plants (Figs 6 and S7). The *Arabidopsis* gene *UFO* and rice *APO1* (orthologs of *TaAPO-A1*) encode for an F-box containing protein. It has been shown that the rice *APO1* and *Arabidopsis UFO* are important for floral development in respective species^19,21^. Molecularly, the proteins SKP1, cullin like, and F-box containing polypeptides form the SCF protein complexes to function as E3-ubiquitin ligases that target specific proteins for degradation^40,41^. For example, it was shown that *Arabidopsis UFO* indirectly regulates the expression of class B floral homeotic gene *APETALA 3* by targeting the degradation of proteins which negatively regulate its transcription^21^. The rice *apo1* mutants show a reduction in the number of primary branches and, thereby, the number of spikelets due to the precocious conversion of inflorescence meristem (IM) to spikelet meristem (SM). Such a mutant phenotype offers an indication that *APO1* might target proteins that promote the precocious conversion of IM to SM for degradation in a functional state. In line with this idea, the dominant gain of function *APO1* alleles with an elevated expression as well as overexpression transgenic lines of *APO1* showed prolonged inflorescence development resulting in more branch iterations and consequently more spikelets^20^.

From our promoter analysis, we found an INDEL where the 115-bp insertion was always associated with high TSN haplotype, whereas the deletion with low TSN haplotype. From this finding, it may be inferred that winter wheat genotypes in the haplogroup with insertion polymorphism have slightly elevated expression of *TaAPO-A1* leading to prolonged maturation of inflorescence meristem and eventually producing more spikelets per spike. Conversely, the deletion haplotype has a comparatively reduced expression level of *TaAPO-A1*, leading to less number of spikelets. Nevertheless, validation of the INDEL haplotype across the whole winter wheat panel as well as expression analysis of *TaAPO-A1* in the two haplogroups with high and low TSN may offer further insights into the regulation of TSN in wheat.

## Conclusions

Our results demonstrate that with the availability of modern genomic tools such as the wheat reference sequence and the access to *The 10*+ *Wheat Genome Project*, the way from phenotype to a candidate gene is shortened considerably. Nevertheless, robust genetic analyses including appropriate mapping populations, accurate and high-density genotyping, and proper phenotypic analyses are prerequisites to detecting significant QTL regions from which the causative genes could be deduced.

## Materials and Methods

### Phenotypic data analyses

The data for total spikelet number (TSN), spike length (SL), and flowering time (FT) were collected on an elite European winter wheat panel comprising of 518 varieties. The whole panel was grown in the experimental fields of Leibniz Institute of Plant Genetics and Crop Plant Research (IPK) Gatersleben, Germany in plots of 2 m^2^ as single replication in three cropping seasons (2015/16; 2016/17; and 2017/18), henceforth called environments. The traits TSN and SL were recorded in two environments (2016/17 and 2017/18) from ten spikes per plot as the total number of spikelets and spike length in centimeters (cm) from basal spikelet to the top of a spike by excluding the awns. The arithmetic mean of TSN and SL from ten spikes were calculated to represent the phenotypic value of traits in the individual environments. Flowering time was recorded in all three environments by counting the number of days from the first of January to when approximately half of the spikes in a plot flowered. The phenotypic data for grain yield estimated in eight environments were taken from the previous study for comparison purposes^23^. A linear mixed-effect model was used for across environment phenotypic data analysis as:

$${y}_{ij}=\mu +{G}_{i}+{E}_{j}+{e}_{ij}$$

where, *y*_*ik*_ is the phenotypic record of the *i*^*th*^ genotype in the *j*^*th*^ environment, *μ* is the common intercept term, *G*_*i*_ is the effect of the *i*^*th*^ genotype, *E*_*j*_ is the effect of the *j*^*th*^ environment, and *e*_*ij*_ denotes the corresponding error term. All effects, except the intercept, were assumed to be random to calculate the individual variance components. The broad-sense heritability (*H*^2^) was calculated as:

$${H}^{2}=\frac{{\sigma }_{G}^{2}}{{\sigma }_{G}^{2}+\frac{{\sigma }_{e}^{2}}{nE}}$$

where, *σ*_*G*_^2^ and *σ*_*e*_^2^ denote the variance components of the genotype and the error, respectively; *nE* denotes the number of environments. To calculate the best linear unbiased estimations (BLUEs), the intercept and the genotypic effects were assumed to be fixed in the above model.

### Genotypic data analyses, population structure, and linkage disequilibrium

All 518 varieties were extensively genotyped with the 35k Affymetrix and 90k iSELECT single nucleotide polymorphism (SNP) arrays^24,25^ which generated in total 116,730 SNP markers (35k = 35,143; 90k = 81,587). Moreover, we genotyped the whole panel with functional markers for the candidate genes such as photoperiodism (*Ppd-D1*), reduced height (*Rht*), and vernalization (*Vrn1*). The quality of the marker data was improved by removing the markers harboring >10% heterozygous or missing calls and markers with a minor allele frequency of <0.05. The mean of both alleles imputed the remaining missing data. The quality control resulted in a total of 39,908 markers, which were used in subsequent analyses.

Population structure based on marker genotypes was examined by principal component (PC) analysis. The first two PCs were drawn to see the sub-clustering among varieties. Furthermore, the genetic relatedness among varieties was evaluated by an additive variance-covariance genomic relationship matrix. To infer the hidden population sub-structuring, an inference algorithm LEA (Landscape and Ecological Association Studies) was used by assuming ten ancestral populations (*K* = 1–10). The function *snmf*, which provides the least-squares estimates of ancestry proportions and estimates an entropy criterion to evaluate the quality fit of the model by cross-validation, was used. The number of ancestral populations best explaining the data can be chosen by using the entropy criterion. We performed ten repetitions for each *K*, and the optimal repetition demonstrating the minimum cross-entropy value was used to visualize clustering among varieties via bar plots^42^.

Linkage disequilibrium (LD), the non-random association of alleles at different loci, was measured as the squared correlation (*r*^2^) among markers. The genetic mapping positions of the markers for both arrays were adopted from the data generated for the International Triticeae Mapping Initiative (ITMI) DH population, as described in Sorrells *et al*.^43^. Although inter and intra-chromosomal LD among the loci varies, genome-wide calculation of LD gives a global estimate about the genetic map distance over which the LD decays in a given population. The genome-wide (global) LD was calculated only from the mapped markers.

### Genome-wide association studies

Genome-wide association studies (GWAS) were performed on data taken from the individual environment and SNPs passing the quality criteria *plus* the functional gene markers. Let *n* be the number of varieties and *p* be the predictor marker genotypes. A standard linear mixed-effect model following Yu *et al*.^44^, was used to perform GWAS as:

$$y=\mu +E\tau +X\beta +Pv+Zu+e$$

where, *y* is the *n* × 1 vector of phenotypic record of each genotype in each environment, *μ* is the common intercept, *τ*, *β*, *v*, *u* and *e* are the vectors of the environment, marker, population (principal components), polygenic background, and the error effects, respectively; *E*, *X*, *P* and *Z* are the corresponding design matrices. In the model, *μ*, *τ*, *β* and *v* were assumed to be fixed while *u* and *e* as random with *u* ~ *N*(0,*Gσ*_*a*_^2^), and *e* ~ *N*(0,*Iσ*_*e*_^2^). The *n* × *n* variance-covariance additive relationship matrix (*G*) was calculated from *n* × *p* matrix *W* = (*w*_*ik*_) of marker genotypes (being 0, 1 or 2) as $G=\frac{{\sum }_{k=1}^{p}({w}_{ik}-2{p}_{k})({w}_{jk}-2{p}_{k})}{2{\sum }_{k=1}^{p}{p}_{k}(1-{p}_{k})}$ where, *w*_*ik*_ and *w*_*jk*_ are the profiles of the *k*^*th*^ marker for the *i*^*th*^ and *j*^*th*^ variety, respectively; *p*_*k*_ is the estimated frequency of one allele in *k*^*th*^ marker, as described by VanRaden^45^.

As population stratification and familial relatedness can severely impact the power to detect true marker-trait association (MTA) in GWAS, different statistical models were used to avoid spurious MTA viz., (1) general linear model (*naive*), (2) population structure correction via principal components (*PCs*), (3) correction of familial relatedness via genomic relationship matrix (*G*), and (4) correction of population structure and relatedness via *PCs* and *G*. It is expected that using both *PCs* and *G* in the model can enhance the accuracy of GWAS. Along with this, environmental fixed effects were assigned in all model scenarios. The models described above were compared by plotting the expected *versus* the observed $-{\log }_{10}(P-{\rm{value}})$ in a quantile-quantile plot and the best model was determined by checking how well the observed $-{\log }_{10}(P-{\rm{value}})$ aligned with the expected.

To declare the presence of MTA, a false discovery rate (FDR) < 0.05 to account for multiple testing was applied^46^. Following Utz *et al*.^47^, the percentage of total genotypic variance (*p*_*G*_) explained by all the QTL passing the FDR threshold was determined as *p*_*G*_ = [*R*_*adj*_^2^/*H*^2^] × 100 where, *R*_*adj*_^2^ was calculated by fitting all the MTA in a multiple linear regression model in the order of ascending *P*-values and *H*^2^ is the broad-sense heritability. The *p*_*G*_ values of individual QTL were accordingly derived from the sum of squares of the QTL (SS_QTL_) in the linear model.

### Candidate gene identification, haplotype analysis by exploiting resources from *The 10+ Wheat Genome Project*, and the KASP marker development

We narrowed-down the QTL region, and BLASTed sequences of all the significant markers present within the genetically defined region onto the physical map of the corresponding chromosome of the reference sequence of the wheat genome which yielded significant physical region^48,49^. Afterward, the gene identifiers (gene-IDs) present within the physical region and their annotated functional descriptions were retrieved. Among them was a most likely candidate gene *TaAPO-A1* for TSN.

*The 10*+ *Wheat Genome Project* is an international collaborative effort that aims to assemble the genomes of more than ten wheat varieties bred in different countries to characterize the wheat pan-genome (http://www.10wheatgenomes.com/). We retrieved the genomic sequence of *TaAPO-A1* for ten wheat varieties from *The 10*+ *Wheat Genome Project* and aligned the sequences to observe the haplotype structures. The SNP that revealed a clear haplotype structure was used to design a Kompetitive Allele Specific PCR (KASP) marker in the candidate gene. The allele-wise phenotypic distribution of the investigated traits with the gene-specific KASP marker was analyzed by plotting the boxplots. The significance (*P-*values) between the mean values of genotypes harboring different KASP marker alleles was determined by two-sided *t*-test. Moreover, we performed a second round of GWAS by incorporating the gene-specific KASP marker in the original SNP matrix to determine whether it associates with the phenotypes. The GWAS parameters were kept the same as described above.

### Multiple sequence alignment and phylogenetic analyses

The TaAPO-A1 protein sequence (corresponding to *TraesCS7A01G481600*) was used as a BLAST query to retrieve the monocot, dicot and Bryophyte orthologs from EnsemblPlants (http://plants.ensembl.org/index.html) and Phytozome *v*12.1 (https://phytozome.jgi.doe.gov/pz/portal.html) databases. The orthologous protein sequences were aligned using ClustalW in Geneious *v*11.0.5^50^. The protein alignment was used to infer a maximum likelihood (ML) phylogeny. The JTT matrix^51^ was identified as the best-fitting model of protein evolution with ProtTest 3^52,53^ and the Akaike Information Criterion (AIC). The evolutionary history among *TaAPO-A1* orthologs across various plant species was inferred using RAxML *v*8.2.12^54^ with PROTGAMMAJTT model, rapid bootstrapping of 100 replicates, and search for best-scoring ML tree (options “-f a -x 1 -# 100”). The consensus tree was further processed to collapse branches with bootstrap support lower than 50%, and the tree was rooted with the Bryophytes *Physcomitrella patens* and *Selaginella moellendorffii* as an outgroup.

## Supplementary information

figs_s1-s7

Table S1

Table S2

Table S3

Table S4

Table S5
