# Supplementary material for: TaAPO-A1, an ortholog of rice ABERRANT PANICLE ORGANIZATION 1, is associated with total spikelet number per spike in elite European hexaploid winter wheat (Triticum aestivum L.) varieties
Source: Sci Rep. 2019 Sep 25;9:13853. doi: 10.1038/s41598-019-50331-9 (PMC6761172; doi:10.1038/s41598-019-50331-9)
Supplement: Supplementary file 1 — figs_s1-s7 [file 41598_2019_50331_MOESM1_ESM.pdf]

Article type: Research article

TaAPO-A1, an ortholog of rice *ABERRANT PANICLE ORGANIZATION 1*, is associated with total spikelet number per spike in elite European hexaploid winter wheat (*Triticum aestivum* L.) varieties

Quddoos H. Muqaddasi[1*], Jonathan Brassac[1], Ravi Koppolu[1], Jörg Plieske[2], Martin W. Ganal[2], and Marion S. Röder[1]

[1]Leibniz Institute of Plant Genetics and Crop Plant Research (IPK), Corrensstraße 3, D-06466 Stadt Seeland, OT Gatersleben, Germany.

[2]TraitGenetics GmbH, Am Schwabeplan 1b, D-06466 Stadt Seeland, OT Gatersleben, Germany.

*Corresponding author: muqaddasi@ipk-gatersleben.de

Supplementary figures

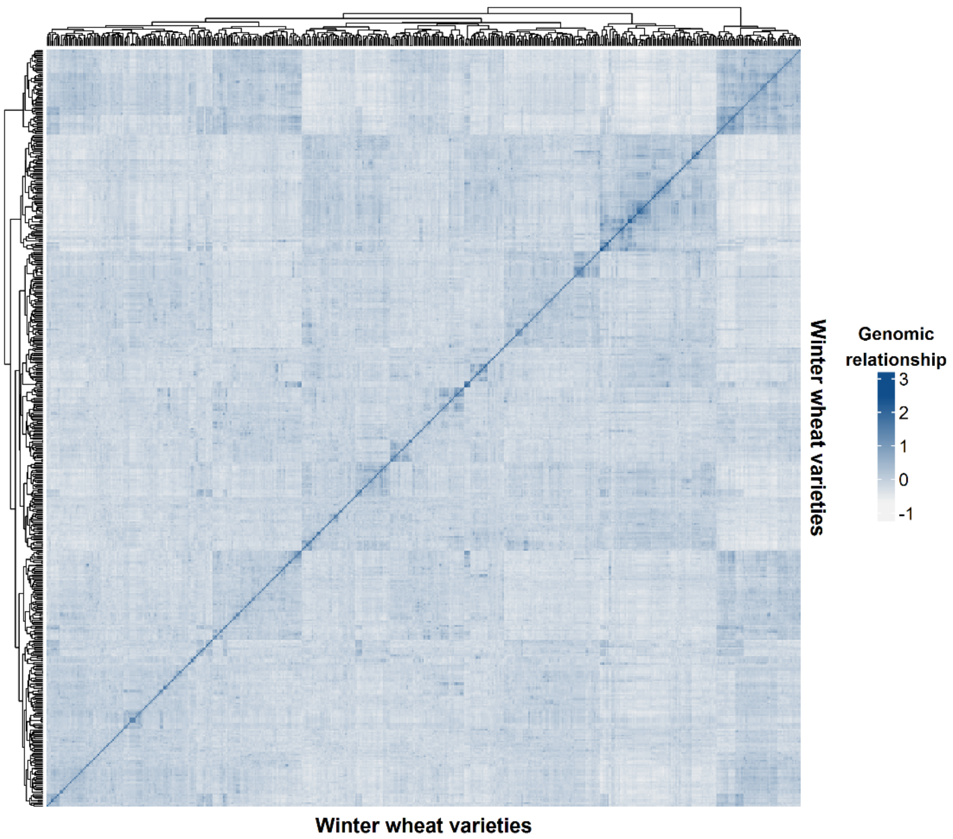

Figure S1. Heat map of the genomic relationships among 518 elite European winter wheat varieties based on 39,908 marker genotypes. The varieties are ordered according to hierarchical cluster analysis. The scale of genomic relationships is given in the figure.

(a)

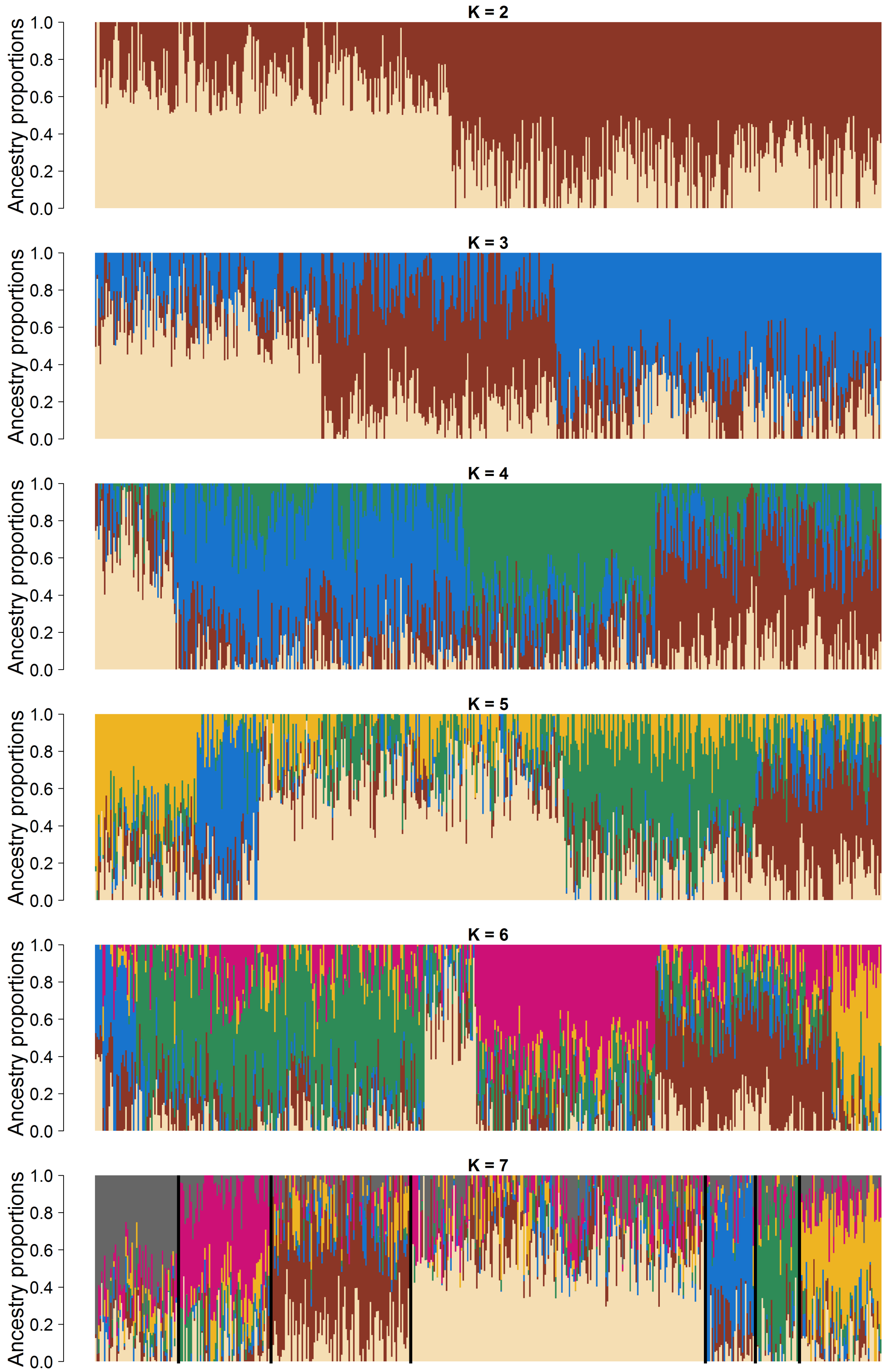

(b)

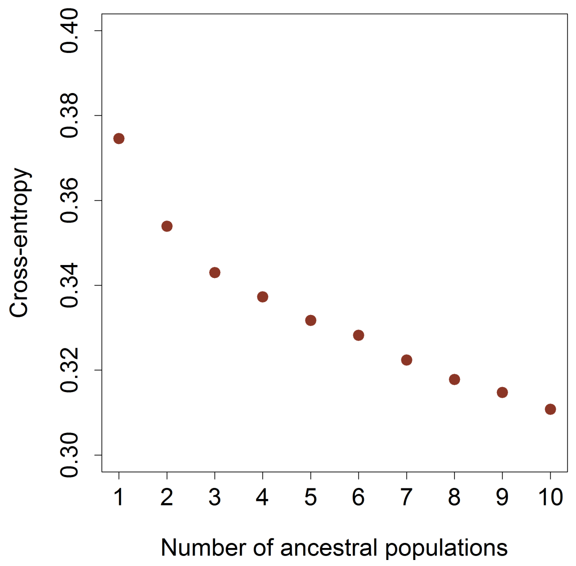

Figure S2. Population structure analysis of the 518 elite European winter wheat varieties based on 39,809 marker genotypes. (a) Bar plots show the existence of admixed sub-populations, (b) The cross-entropy plot shows that there exists a minimal sub-structuring in the panel.

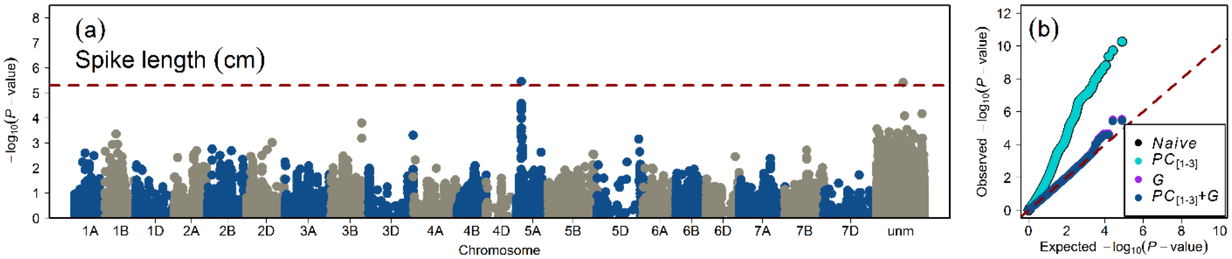

Figure S3. Summary of the genome-wide association studies (GWAS) of spike length in the population of 518 elite European winter wheat varieties. (a) Manhattan plot shows the distribution of markers significance $-\log_{10}(P-\text{value})$ along the chromosomes. The correction for population stratification and familial relatedness was performed by using the first three principal components ($PC_{[1-3]}$) and an additive genomic relationship matrix (*G*) in a linear mixed-effect model. The red dashed line marks the multiple testing criteria of false discovery rate (FDR) <0.10, **(b) Quantile-quantile plot showing the distribution of observed versus expected (red dashed line) $-\log_{10}(P-\text{value})$.** The naïve model represents the GWAS without the correction of population structure, the $PC_{[1-3]}$ model represents the population structure corrected with the first three *PCs*, the *G* model represents the familial relatedness corrected with a genomic relationship matrix, and the $PC_{[1-3]}+G$ model represents the population structure and familial relatedness corrected with the *PCs* and the *G* matrix. The color code for different models is given in the figure legend.

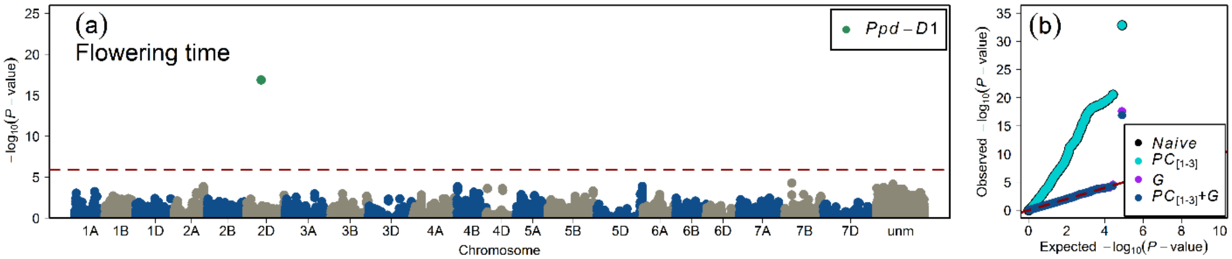

Figure S4: Summary of the genome-wide association studies (GWAS) of flowering time in the population of 518 elite European winter wheat varieties. (a) Manhattan plot shows the distribution of markers significance $-\log_{10}(P-\text{value})$ along the chromosomes. The correction for population stratification and familial relatedness was performed by using the first three principal components ($PC_{[1-3]}$) and an additive genomic relationship matrix (*G*) in a linear mixed-effect model. The red dashed line marks the multiple testing criteria of false discovery rate (FDR) <0.05, **(b) Quantile-quantile plot showing the distribution of observed versus expected (red dashed line) $-\log_{10}(P-\text{value})$.** The naïve model represents the GWAS without the correction of population structure, the $PC_{[1-3]}$ model represents the population structure corrected with the first three *PCs*, the *G* model represents the familial relatedness corrected with a genomic relationship matrix, and the $PC_{[1-3]}+G$ model represents the population structure and familial relatedness corrected with the *PCs* and the *G* matrix. The color code for different models is given in the figure legend.

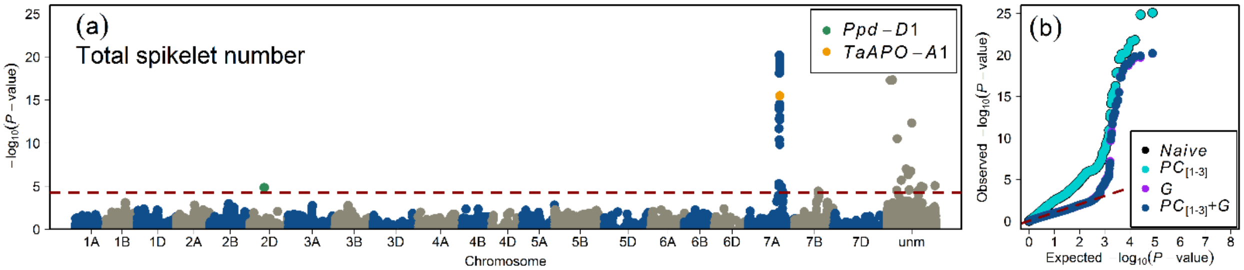

Figure S5. Summary of the genome-wide association studies (GWAS) of total spikelet number per spike in the population of 518 elite European winter wheat varieties. (a) Manhattan plot shows the distribution of markers significance $-\log_{10}(P - \text{value})$ along the chromosomes. The correction for population stratification and familial relatedness was performed by using the first three principal components ($PC_{[1\text{-}3]}$) and an additive genomic relationship matrix (*G*) in a linear mixed-effect model. The red dashed line marks the multiple testing criteria of false discovery rate (FDR) <0.05, **(b) Quantile-quantile plot showing the distribution of observed versus expected (red dashed line) $-\log_{10}(P - \text{value})$.** The naïve model represents the GWAS without the correction of population structure, the $PC_{[1\text{-}3]}$ model represents the population structure corrected with the first three *PCs*, the *G* model represents the familial relatedness corrected with a genomic relationship matrix, and the $PC_{[1\text{-}3]}+G$ model represents the population structure and familial relatedness corrected with the *PCs* and the *G* matrix. The color code for different models is given in the figure legend.

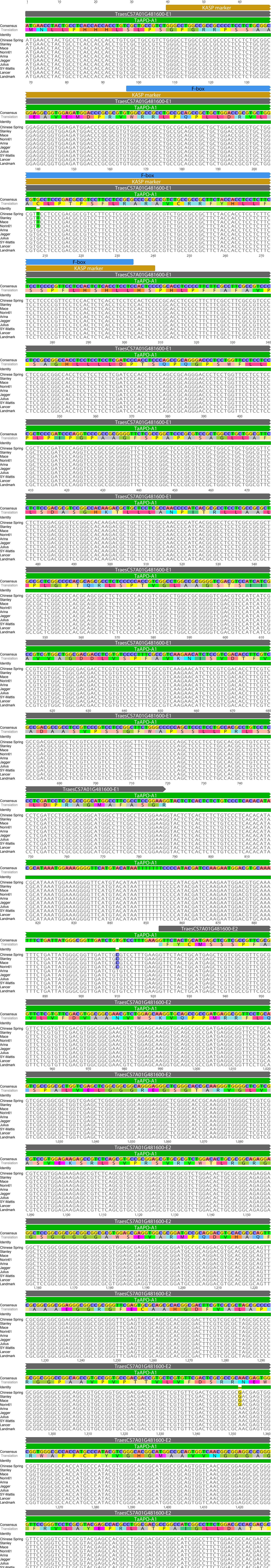

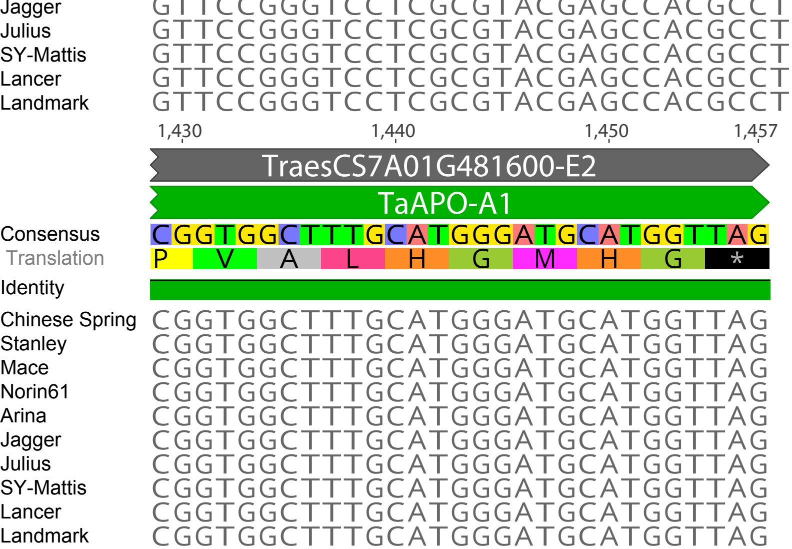

Figure S6. DNA alignment of the *TaAPO-A1* gene across ten wheat varieties.
The DNA sequences of all ten varieties were taken from the wheat reference sequence *v*1.0 and *The 10+ Wheat Genome Project*. The location of the F-box and the KASP marker for *TaAPO-A1* gene is indicated with blue and brown annotations, respectively. The threshold for the consensus calling is 50% matches and mismatches to the consensus are highlighted in the alignment.

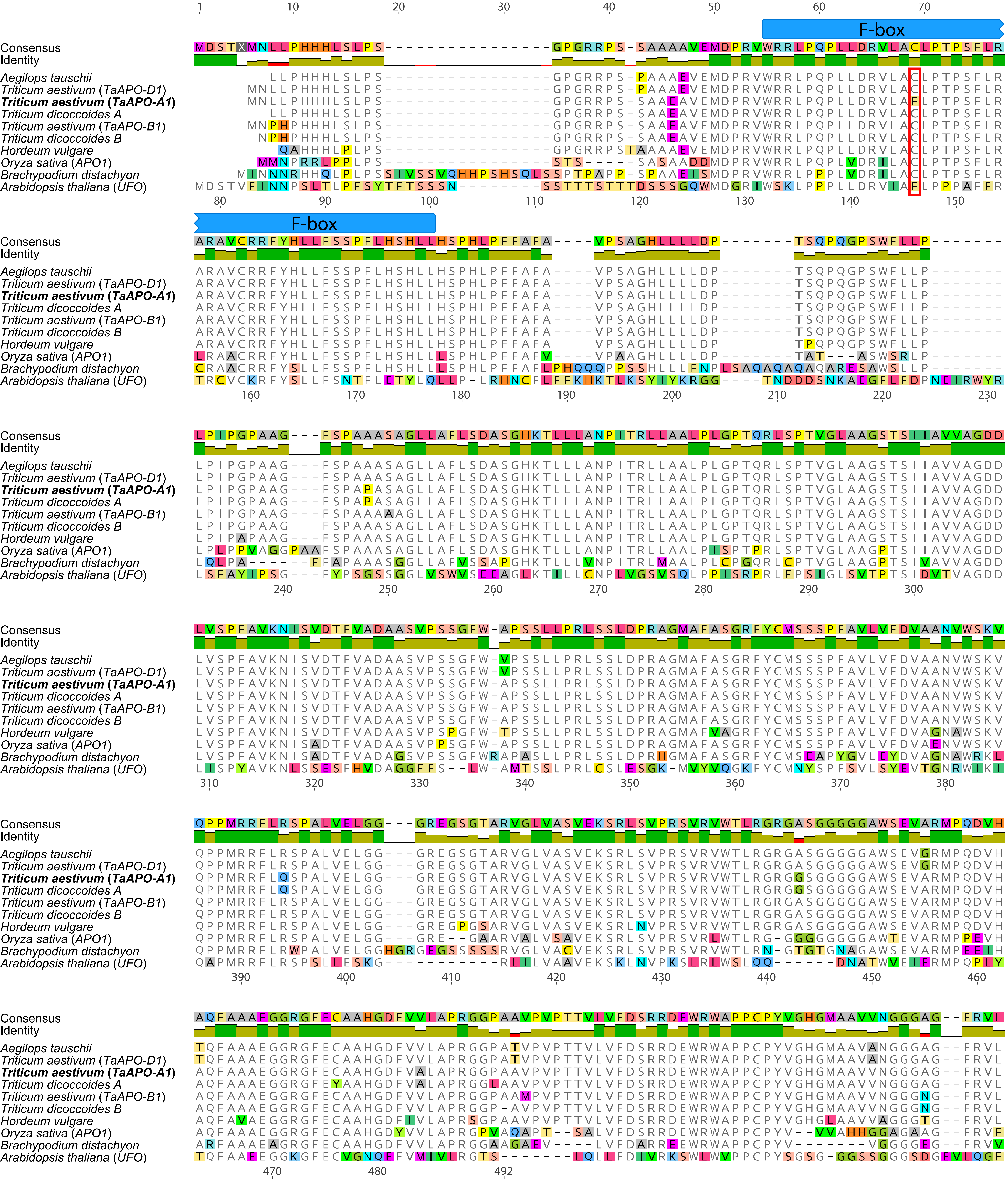

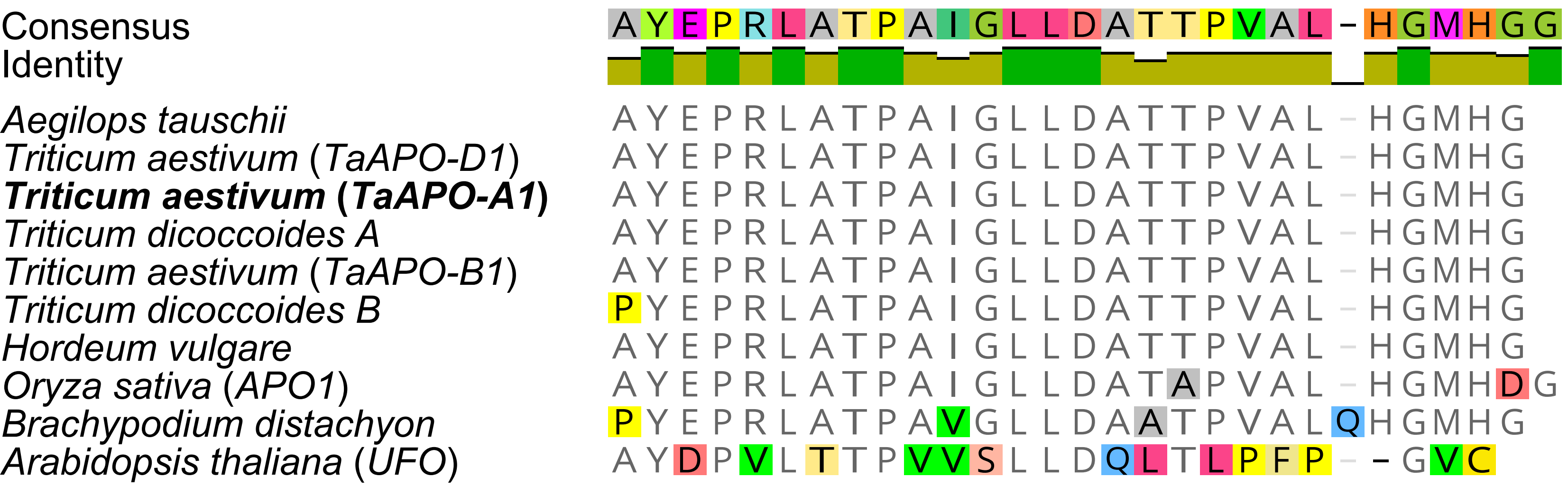

Figure S7. Protein alignment of the TaAPO-A1 orthologs across different plant species.
The protein sequences were taken from EnsemblPlants. The F-box is annotated in the blue. The non-synonymous mutation (caused by G/T base substitution) is highlighted in red frame. The threshold for the consensus calling is 50% matches and mismatches to the consensus are highlighted in the alignment.
